# Pharma-cartography: Navigating the complexities of antibiotic supply to rural livestock in West Bengal, India, through value chain and power dynamic analysis

**DOI:** 10.1371/journal.pone.0281188

**Published:** 2023-02-02

**Authors:** Mathew Hennessey, Ayako Ebata, Indranil Samanta, Ana Mateus, Jean-Christophe Arnold, Dominic Day, Meenakshi Gautham, Pablo Alarcon

**Affiliations:** 1 Veterinary Epidemiology, Economics and Public Health Group, Department of Pathobiology and Population Sciences, Royal Veterinary College, London, United Kingdom; 2 Institute of Development Studies, Brighton, United Kingdom; 3 Department of Veterinary Microbiology, West Bengal University of Animal and Fishery Sciences, Kolkata, India; 4 Department of Global Health and Development, Faculty of Public Health and Policy, London School of Hygiene and Tropical Medicine, London, United Kingdom; University of Liverpool & International Livestock Research Institute (ILRI), UNITED KINGDOM

## Abstract

Antibiotic resistance threatens provision of healthcare and livestock production worldwide with predicted negative socioeconomic impact. Antibiotic stewardship can be considered of importance to people living in rural communities, many of which depend on agriculture as a source of food and income and rely on antibiotics to control infectious diseases in livestock. Consequently, there is a need for clarity of the structure of antibiotic value chains to understand the complexity of antibiotic production and distribution in community settings as this will facilitate the development of effective policies and interventions. We used a value chain approach to investigate how relationships, behaviours, and influences are established during antibiotic distribution. Interviews were conducted with key informants (n = 17), value chain stakeholders (n = 22), and livestock keeping households (n = 36) in Kolkata, and two rural sites in West Bengal, India. Value chain mapping and an assessment of power dynamics, using manifest content analysis, were conducted to investigate antibiotic distribution and identify entry points for antibiotic stewardship. The flow of antibiotics from manufacturer to stockists is described and mapped and two local level maps showing distribution to final consumers presented. The maps illustrate that antibiotic distribution occurred through numerous formal and informal routes, many of which circumvent antibiotic use legislation. This was partly due to limited institutional power of the public sector to govern value chain activities. A ‘veterinary service lacuna’ existed resulting in livestock keepers having higher reliance on private and informal providers, who often lacked legal mandates to prescribe and dispense antibiotics. The illegitimacy of many antibiotic prescribers blocked access to formal training who instead relied on mimicking the behaviour of more experienced prescribers–who also lacked access to stewardship guidelines. We argue that limited institutional power to enforce existing antibiotic legislation and guide antibiotic usage and major gaps in livestock healthcare services make attempts to curb informal prescribing unsustainable. Alternative options could include addressing public sector deficits, with respect to both healthcare services and antibiotic provision, and by providing resources such as locally relevant antibiotic guidelines to all antibiotic prescribers. In addition, legitimacy of informal prescribers could be revised, which may allow formation of associations or groups to incentivise good antibiotic practices.

## 1. Background

Antibiotic stewardship is an important tool to mitigate the effects of antibiotic resistance (ABR) [1, 2], which threatens the provision of effective healthcare and livestock production worldwide and has direct social and economic consequences [3]. Antibiotic stewardship has been defined as promotion of “optimal selection, dosage, and duration of [antibiotic] treatment that results in the best clinical outcome for the treatment or prevention of infection, with minimal toxicity to the patient and minimal impact on subsequent resistance” [4]. Current efforts to improve antibiotic stewardship often focus on strengthening regulation, knowledge, and awareness regarding antibiotic use and ABR [5–7]. However, in many low and middle-income countries (LMICs), where legislation for human and veterinary medicine products and enforcement capacity and access to diagnostic infrastructure is limited [8, 9], implementing effective stewardship remains challenging.

Historically, antibiotics have been used during animal production to protect against and treat disease and to promote livestock growth. While recent trends for Asia, the Far East and Oceania have shown a decrease in overall usage per kilogram of animal biomass (230mg/kg in 2016 to 150mg/kg in 2018) 36% of countries in this region still report the use of antibiotics for growth promotion [10]. India is one such country and is currently experiencing substantial agricultural sector growth [11] due to increasing disposable incomes creating demand for meat [12] and a large export industry [13]. Due to the high level of non-therapeutic use and misuse documented in India’s agriculture and aquaculture systems [14–16], antibiotic use in India’s agriculture is concerning. Consequently, agricultural growth contributes to an already high level of antibiotic use in India [17].

By Indian regulations, antibiotics can only be sold on the prescription of a qualified medical practitioner, but their over the counter sales (i.e., without a prescription) continue in both human and animal health, and by providers who do not have formal professional qualifications [18–25]. This raises questions about the supply systems through which antibiotics reach communities mainly through non-formal providers. Currently, there is little known about the structure of antibiotic value chains in India [26, 27] and a need to improve clarity to develop effective policies and interventions.

To date, there have been few documented attempts in India or other LMICs to implement antibiotic stewardship at a national level in livestock [28, 29], and a lack of interventions which consider all stakeholders involved with the production and distribution of antibiotics [28]. Consequently, the aim of this study was to analyse the antibiotic value chain for livestock in rural West Bengal and identify ways to foster antibiotic stewardship in the agricultural sector. Specifically, we sought to achieve the following objectives; (1) to map value chains from manufacturer to end users, (2) to analyse the governance structure and power dynamics along the value chain, and (3) identify effective entry points for antibiotic stewardship interventions. The study presented here is part of the larger One Health Antibiotic Stewardship in Society research project (OASIS; www.oasisamr.com) that investigates the patterns and drivers of antibiotic use in human and livestock healthcare in rural community settings in West Bengal.

## 2. Methods

For this study, we use a value chain analysis approach which assesses the systems in which commodities are produced and exchanged, and attempts to detail the often complex relationships which occur within and between nodes–the places or actors—along the value chain [30–33]. Consequently, this approach has potential to help identify the factors influencing behaviours of actors along antibiotic value chains and therefore aid the development of antibiotic stewardship interventions.

### 2.1 Study area

West Bengal was chosen as a case study as it is one of India’s major agricultural states—milk, egg, and meat contribute to 3%, 8%, and 10% of national production respectively [34]–where rural people often rely on livestock production for food and income [35] and has been identified as a global hotspot for antibiotic resistance in animals [36].

Two differing Gram Panchayats [a village administrative unit] in South 24 Parganas district, were chosen from those known to have numerous medical informal providers in the area and in a purposive manner based on the number of households keeping livestock (50% and 90% of households reported to keep livestock in sites 1 and 2 respectively [37]) and the distance from Kolkata (60km south-west and 95km south-east for sites 1 and 2 respectively). These criteria were selected due to the hypothesis that the concentration of livestock keeping households and proximity to Kolkata would affect the structure of the antibiotic value chains. The reason for considering the number of medical informal providers in the sites was due to the hypothesis that there may be overlaps between the human and livestock antibiotic provision landscapes, a phenomenon known to have occurred in some LMICs [38–43], but that at the time of this study, had not yet been documented in India.

### 2.2 Data collection

**Fig 1:**
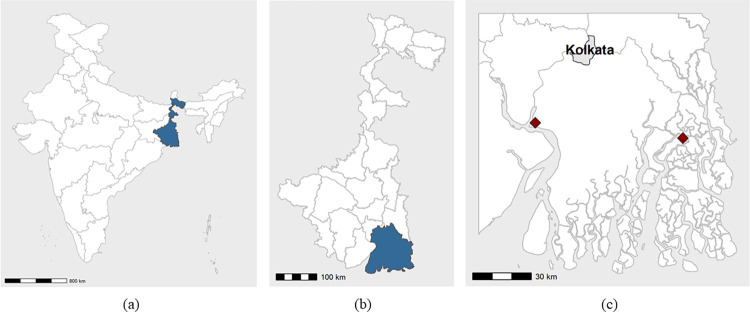
Maps showing; a) West Bengal within India, b) South 24 Parganas within West Bengal, and c) site 1 (west) and site 2 (east) within South 24 Parganas (Created in R Studio ver. 2022.07.1; data sources [55, 56]).

Interviews were conducted with key informants (KIs; n = 17)–individuals with anticipated high-level knowledge of the entire antibiotic value chain, antibiotic value chain stakeholders–individuals who were questioned primarily about their own practice, (SHs; n = 22), and with livestock keeping households (LKs; n = 36). Interviews took place in Kolkata, and the two study sites between June 2019 and January 2020 (Table 1; Fig 1).

**Table 1:** Summary of key-informant, stakeholder, and household interviews.

	Kolkata	Site 1	Site 2
Key-informant interviews	Drug control department official (n = 1) Pharmaceutical representatives (n = 3) Pharmacology academic (n = 1) Private vets (n = 2) Experienced wholesaler (n = 1)	Ex-government veterinarian (n = 1) Ex-village chief (n = 1) Teacher (n = 2)	NGO veterinarian (n = 1) Block livestock development officer (n = 1) Block veterinary officer (n = 1) Mobile veterinary officer (n = 1)
Stakeholder interviews		Veterinary medicine representative (n = 1) Veterinary drug shop owner (n = 2) Homeopath (human health) (n = 3) Para-vet (n = 1) Pranibandu (n = 1) Animal development volunteer (n = 1) Human informal provider (n = 4) Human drug shop owner (n = 2)	Veterinary medicine representative (n = 1) Poultry shop owner (n = 1) Drug wholesalers/retailers (n = 2) Para-vets (n = 2) Pranimitra (n = 1) Pranibandu (n = 1)
Household interviews		n = 22	n = 14

Interviewees were selected using purposive convenience sampling using a network of partners in the project and a snowballing technique with the aim of capturing perspectives from all actors operating in the value chain. In Site 1, an ex-village chief, teachers, and ex-government veterinarian were identified as key informants and interviewed to provide an overview of the area and to help identify livestock keepers and livestock healthcare providers. In Site-2, we followed the same process by interviewing a non-government organisation (NGO) veterinarian and veterinary and livestock development officers working for the public sector. In both sites, as many as possible of the identified stakeholders operating in the system were contacted and invited to participate in an interview. Households in both sites were selected to provide information on a range of livestock types (e.g., cows, goats, sheep, chickens) and production systems (e.g., commercial and subsistence purposes). Information gathered from a review of existing literature was used to generate *a priori* interview guides for the semi-structured in-depth interviews. These guides were designed to differentiate the types of stakeholders involved in the antibiotic value chain, their roles, inter-actor relationships, and links between livestock and human antibiotic value chains. Where possible interviewees were asked to provide information about which antibiotics they most commonly dealt with, and their perceptions of ABR. When participants were unfamiliar with the term ‘antibiotics’, for example some of the livestock keepers, we asked broader questions about treatments and general medicines to delineate their knowledge. Complete interview guides are provided in S1–S3 Files, along with a summary of topics in Table 2.

**Table 2:** Summary of topics covered in interview guides.

	Key topic	Objective
Key-informant & Stakeholders	1. Role of the participant	1. To contextualise the informant’s perspective
	2. Value chain mapping	2. Mapping exercise; detailing all antibiotic producers, providers, and users
	3. Antibiotic quality	3. Understanding where variations in quality exist within the chain
	4. Governance	4. Delineating the mechanisms which influence value chain actor’s behaviour
	5. Crossover antibiotic use	5. Human antibiotics being used in livestock and vice versa
Livestock keeping households	1. Demographic details	1. Type, number, and reasons for keeping livestock
	2. Animal health care	2.Type of animal health care provider used and drivers for their use.
	3. Overlaps in antibiotic use	3. Human antibiotics being used in livestock and vice versa

Questions were open-ended to minimise interviewer confirmation bias and interviewee social desirability bias [44]. Interviews were conducted in either English or Bangla and, when consent granted, voice recorded. A member of the local research team acted as a translator where necessary.

During each interview, key-informants and stakeholders were asked to help produce antibiotic flowchart diagrams by identifying and characterising all nodes and flows of antibiotics known to them. The characterisation of nodes was completed by prompting interviewees with questions relating to the differences in suppliers, clients and users of antibiotics, and how these nodes are connected. In addition, participants were asked to indicate where the major volume flows of antibiotics occurred. During household interviews, information was gathered about how drivers for choice of healthcare provider relates to the antibiotic value chain.

### 2.3 Ethics approval and consent to participate

Ethical approval for the study was obtained by Observational and Interventions Research Ethics Committee and MSc Research Ethics Committee of the London School of Hygiene and Tropical Medicine (Ref: 17484, 03/09/2019 and 16677, 05/06/2019), the Institutional Animal Ethics Committee of West Bengal University of Animal and Fishery Sciences (Ref: IAEC/190(XVII)/B/1288, 19 March 2019), and the Social Sciences Research Ethical Review Board of the Royal Veterinary College (Ref: SR2019-0268, 15/05/2019). All participants involved in this study were provided with an information sheet prior to interview informing that their anonymised data could be used in research publications. Each participant was asked to provide their consent using a written consent form.

### 2.4 Data management and analysis

Interviews conducted in English were transcribed by the primary author while interviews conducted in Bangla were translated and transcribed by a member of the local research team. Data extracted from the transcribed interviews were entered into QRS International NVivo (ver. 12.6.0.959) for data management. Transcripts were created using an intelligent verbatim method–a word for word account of the interview without inclusion of all grammatical errors, mistakes, and expressions of emotion [45].

#### 2.4.1 Antibiotic value chain mapping

In the first part of the analysis we applied the heuristic principles of value chain analysis to map the chains and describe the value chain structure. Following the process indicated in Alarcon et al. [32], we identified nodes and node-interactions. These data were combined with the antibiotic flow diagrams created during each interview to generate three detailed value chain maps that capture the overall system and the diversity of antibiotic providers. The first map presented captures the manufacturing and initial distribution flows of the chains that are common to both study sites while the subsequent two maps capture the locally specific flow of antibiotics in each site. Those nodes reported to be similar were grouped together and key differences noted. The magnitudes of the antibiotic flows were presented qualitatively, based on the participant estimation, using thin to wide arrows.

#### 2.4.2 Value chain power dynamics

Power analysis is critical in understanding how health-related policies lead to inequal access to health care and identify entry points to improve health outcomes particularly by marginalised populations [46]. In our study, we focus on analysing power dynamics in value chains, following the framework developed by Dallas et al. [47]. The framework describes power through two facets. In the first, power is described in terms of the type of nodes involved, being either dyadic—occurring between two nodes—or collective—occurring between a group of nodes. In the second, power is described through the way it exerts influence and is either direct—being focused and having an obvious source and target—or indirect–occurring within a group of nodes due to changes in social structure and collective actions. The result of these variables suggests four types of power (Table 3); 1) bargaining, 2) institutional, 3) demonstrative, and 4) constitutive, though Dallas et al. [47] note the four are not mutually exclusive and some power dynamics may share multiple aspects.

**Table 3:** A typology of power in value chains.

	Direct	Indirect
**Dyadic**	*Bargaining power*	*Demonstrative Power*
	The most common type of power found in value chains and integral to the relationship between firms and actors (such as buyers and suppliers). The degree of bargaining power is proportional to the power asymmetry present in the relationship.	When certain actors establish new standards or production requirements, this may have the effect of influencing others who must adapt their activities in order to remain competitive. This can be considered an extension of *bargaining power* as actors require leverage over others to ensure their new standards are adopted.
**Collective**	*Institutional Power*	*Constitutive Power*
	Power exerted by the combined actions of a collection of actors who organise to create sets of rules or guidelines for how the value chains should operate. Collectives may be internal to the value chain, e.g. industry associations, or partially external such as public institutions and governments.	Power exerted through the combined actions of a collection of actors who are not formally organised. Instead social or consumer movements exert influence on value chain conduct through broadly accepted norms, conventions, expectations, and best practices. Over time these practices may become codified and evolve into institutional power.

Source: Adapted from Dallas et al. [47]

Investigation of power relationships in the value chain provides an opportunity to consider the way actors interact with unintended consequences–or externalities–resulting from chain activities, an area largely absent from early value chain governance literature–e.g., Gereffi et al. [48], Kaplinsky and Morris [30] and Humphrey and Schmitz [49]. This phenomenon is well documented in research on, for instance, labour standards and working conditions where international NGOs, consumer groups, media and labour rights agencies influence value chain actors to adhere to sound working conditions along apparel global value chains [47]. Such evidence indicates that to understand value chain actors’ behaviours and rationales behind them, research needs to address the multiple kinds of influence and power, and the way these affect value chain actors activities [50]. An example of the way value chain work has been used to address sectors with negative health externalities is the tobacco industry. Goger et al.’s [51] examination of changing global tobacco value chains raises concerns over the potential negative impact of decreasing demand on small producers in tobacco dependent countries [51]. Historically, interventions to protect tobacco producers’ interests have primarily focused on crop substitution, but these have not always been successful. The authors argue that governance structures, institutional capacity building, and regulatory initiatives–i.e., the institutional power in the value chain–need to be considered to improve the success of tobacco alternatives for small producers.

Manifest content analysis was used to apply the power dynamic framework [47] to our interview data to identify factors contributing to antibiotic misuse and potential entry points for antibiotic stewardship. This type of qualitative analysis describes a structured, and detailed, approach towards descriptive accounts of interview data [52, 53] through a process of familiarisation, coding, and code restructuring to categorise data into groups and topics. Here, elements within participants responses are taken at having meaning at face value [52], i.e., a *manifest* approach, and are organised according to our study objectives and the power dynamic framework. Consequently, the interpretation of these descriptive findings occurs during our discussion. This is in contrast to other qualitative data analysis techniques, such as thematic analysis, which seek to provide explanation and interpretation through the act of generating themes [54] and is more applicable when datasets are derived from groups of similar individuals.

The primary author became familiar with the data by listening to and transcribing those interviews conducted in English, and by carefully reading all interview transcripts. Data coding began deductively based on topics from the interview guide (Table 2 & S1–S3 Files) and the power dynamics framework, after which codes were discussed with other co-authors experienced in qualitative analysis (PA, MG, AE). An initial codebook was developed and applied to transcripts, with additional codes being developed iteratively and inductively, i.e., a blended approach of deductive and inductive coding was utilised. Topic summaries were generated by combining individual codes and discussed between the research team during numerous group meetings. During analysis we took a critical realist experiential position to interpret the data [54]. Here, participants’ reported experiences of the antibiotic value chain are seen as containing ‘truth’ i.e., an experiential epistemological position.

## 3. Results

### 3.1 Antibiotic value chain

Antibiotic manufacture and distribution to wholesalers, sub-stockists, and retailers.

**Fig 2:**
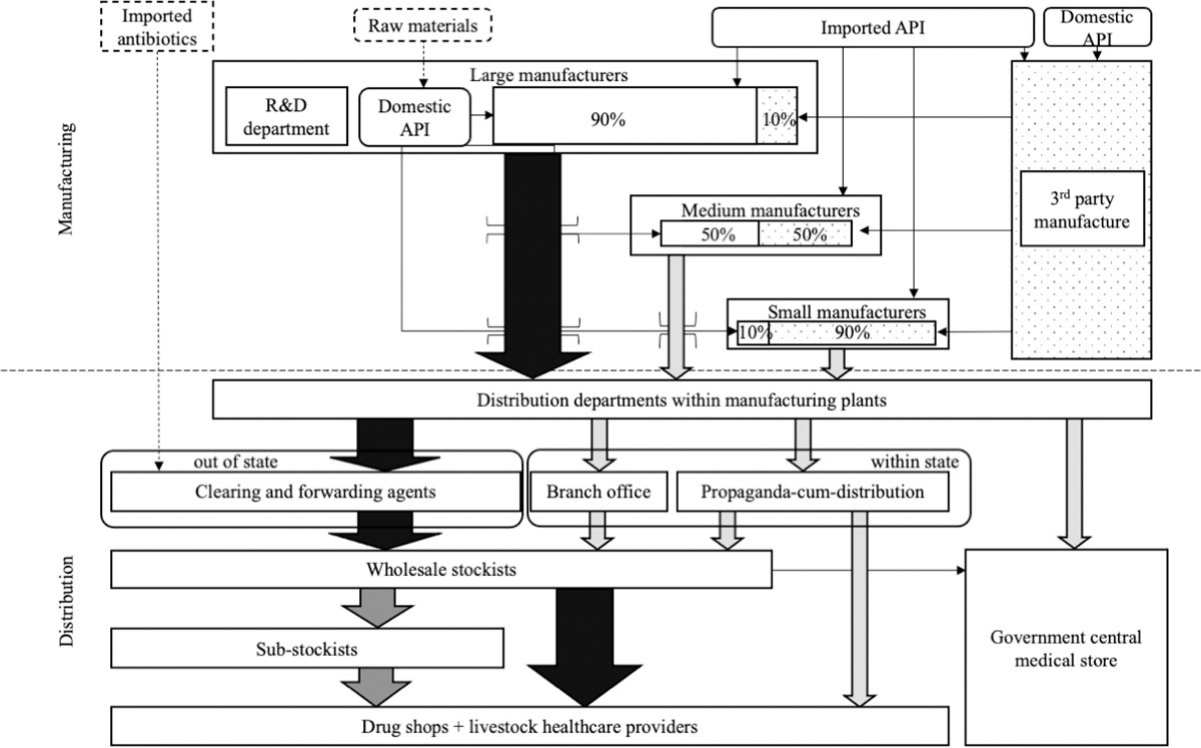
Antibiotic value chain map for manufacture and initial distribution. NB arrow width and shading indicate perceived relative share of antibiotic distribution.

The value chain map for this segment of the chain is shown in Fig 2, for both the public sector and the private sector. In West Bengal, public sector livestock healthcare and antibiotic provision occurs through government employed veterinarians and livestock development assistants, and government trained livestock healthcare workers—known locally as Pranibandhu and Pranimitra. Livestock keepers pay a registration fee (INR 5 for cattle, INR 2 for goats and sheep) to access public services after which they do not pay for services. Private sector provisions occurred through veterinarians, para-vets and other businesses (such as veterinary pharmacies) working outside of the public sector. Here, livestock keepers pay providers for treatments and medicines.

The major flow of antibiotics used in livestock in the study sites originated from domestic companies, distributed from manufacturers to retailers either via independent clearing and forwarding agents (the predominant route) or manufacturing plant branch offices to wholesaler stockists. Wholesale stockists then supplied antibiotics to sub-stockists or direct to retail pharmacies.

Large domestic manufacturers had their own research and development facilities with some producing their own active pharmaceutical ingredients. Most manufacturers outsourced a proportion of production to third party manufacturers–operating on loan licences. Smaller promotion-cum-distribution companies procured generic antibiotics from third party manufacturers before labelling them as branded generics to be sold within state.

Antibiotics were distributed to the public livestock healthcare sector through a central medical store, who obtained antibiotics through a tender process from wholesale stockists.

In both sites numerous representatives of pharmaceutical companies and wholesale drug companies–known locally as ‘medical reps’ or ‘MRs’ facilitated the movement of antibiotics through the value chain. Representatives helped to overcome some of the logistical challenges of moving antibiotics into remote locations, while MRs from pharmaceutical companies also provided antibiotic prescribers with information on how to use antibiotics, updates on new products, and reported back to companies with data on antibiotic performance and procurement challenges.

Antibiotic value chain from stockists to livestock.

**Fig 3:**
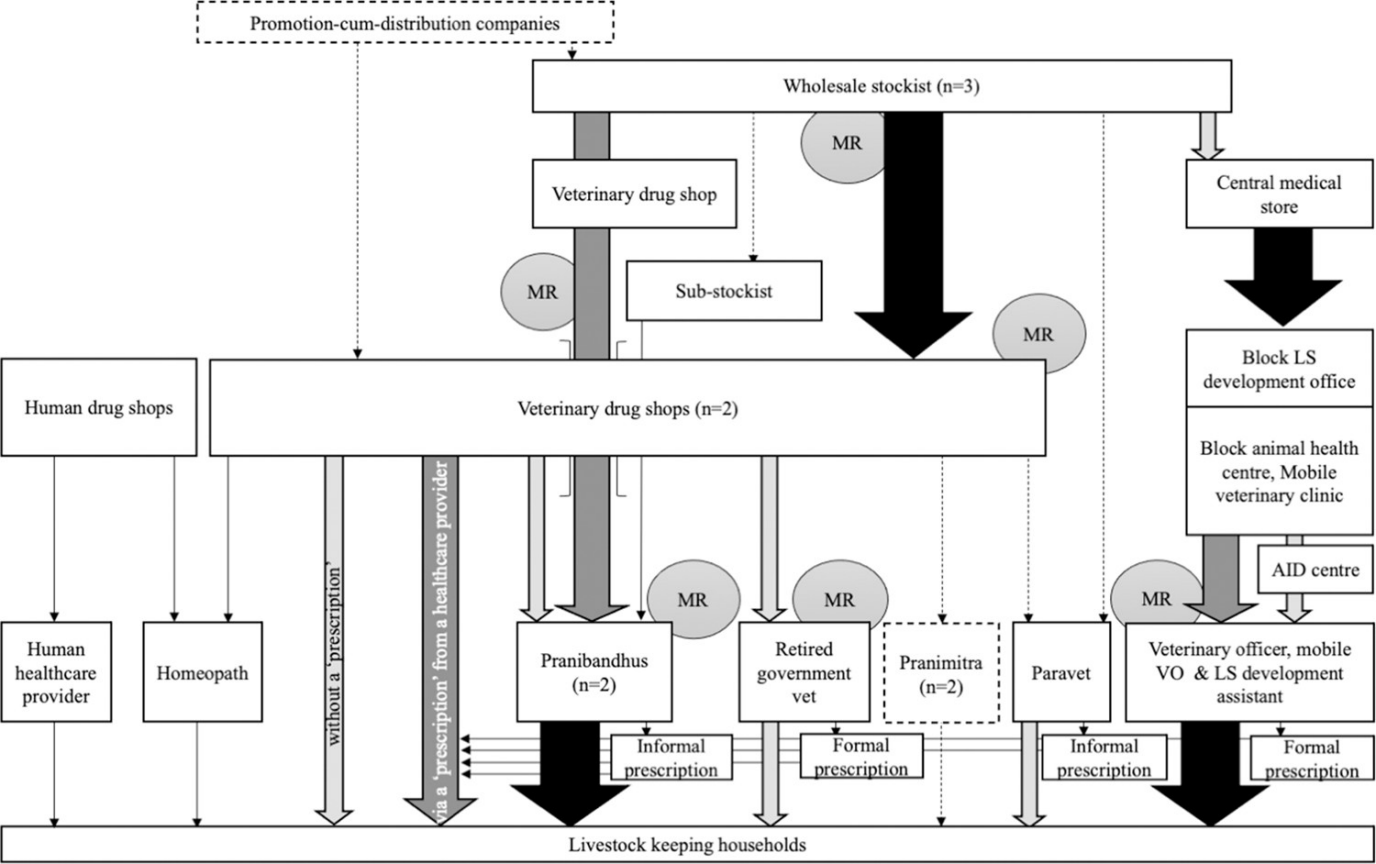
Livestock antibiotic value chain in Site 1. NB arrow width and shading indicate perceived relative share of antibiotic distribution; MR = Medical representative, LS = Livestock, VO = Veterinary Officer.

**Fig 4:**
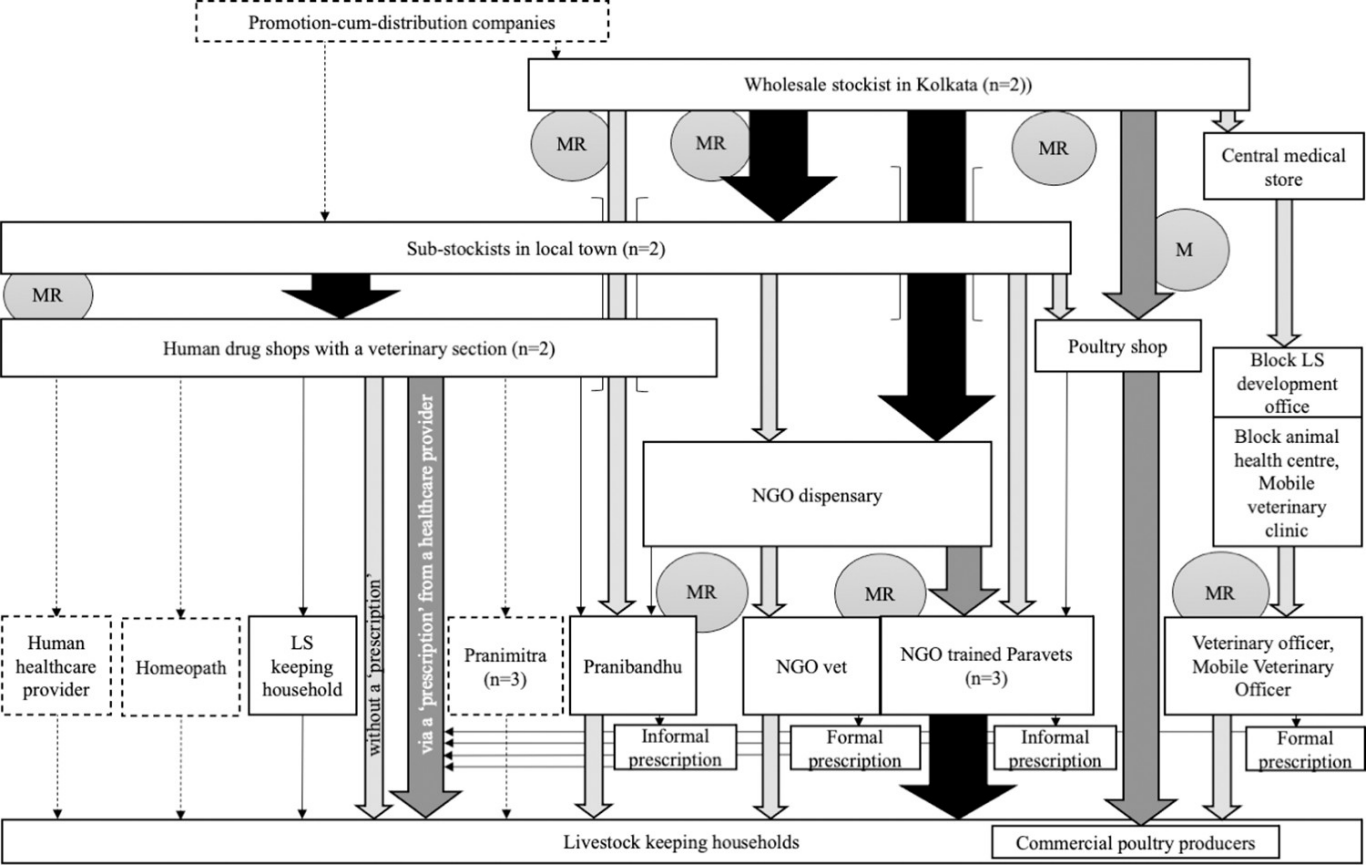
Livestock antibiotic value chain in Site 2. NB arrow width and shading indicate perceived relative share of antibiotic distribution; MR = Medical representative, LS = Livestock, VO = Veterinary Officer.

The antibiotic value chain maps for Site 1 and 2 are shown in Figs 3 and 4, respectively.

Distribution of antibiotics within the public sector was similar in both sites. Antibiotics were delivered to a centre at the administrative block level (below that of subdivision and above the village Gram Panchayats administrative unit)—the Block Livestock Development Office—from the central medical store before being sent to the main livestock healthcare facility—the Block Animal Health Centres, and in site-1 to an additional smaller government livestock site, called an Aid Centre. After interacting with a government veterinarian (either directly, or indirectly through a livestock development assistant) antibiotics were supplied to livestock keepers at no cost to themselves other than the nominal registration fee paid to use the public centres, plus the cost of transport of getting to the animal health centres and Aid centres. However, public sector veterinarians reported frequent antibiotic stock-outs which resulted in livestock keepers having to purchase antibiotics–via a prescription written by the public sector veterinarians- from private sector drug shops.

In the private sector, antibiotic distribution occurred through a variety of routes. In Site 1, most antibiotics were supplied through two veterinary pharmacies in the nearby town; one run by an ex-government veterinarian and his son, the other by a former farmer. In Site 2, the NGO acted as a veterinary pharmacy, supplying antibiotics to livestock via the private formal veterinarian who managed the NGO’s livestock support services (but was only present at weekends) and several para-vets who had been trained by the NGO. Here, para-vets were able to contact the private veterinarian by telephone to ask advice on clinical cases. Antibiotics were also supplied to livestock in Site 2 through two human drug stores and a poultry shop in the adjoining town. Both human drug stores had a small veterinary section but did not employ anyone with livestock or veterinary experience. The poultry shop was managed by a person with experience of raising poultry and supplied antibiotics, as well as other poultry inputs, to the small-scale commercial poultry enterprises operating in the area. In both sites, antibiotics could be purchased either via a prescription or supplied over the counter under the guidance of the pharmacy and drug shop owners. Prescriptions and antibiotics could be supplied formally by the few veterinarians operating in the area or informally by the numerous other livestock healthcare providers operating in the area, including the public-private Pranibandhu and Pranimitra, private sector para-vets, and those informal providers of human healthcare. We defined informal antibiotic supply as occasions when it occurred through actors who lacked a legal mandate to prescribe or dispense antibiotics to livestock (Table 4).

**Table 4:** Description of livestock healthcare providers in the two study sites.

Category	Name	Description	Mandate to prescribe antibiotics [22]	Mandate to medicate livestock [22]
Public sector	Veterinary officer, mobile veterinary officer	Individuals with veterinary qualifications working for the government	Yes	Yes
	Livestock development assistant	Individuals who have completed a two-year diploma in veterinary pharmacy from the government or training institution	No	Yes (Under the supervision of a veterinarian)
Dual public-private	Pranibandhu	Individuals who have received six-month government training in artificial insemination and first aid but who also work in a private capacity to provide livestock healthcare	No	No
	Pranimitra	Individuals who have received 15-day training in vaccination and first aid and infrequently provide private livestock healthcare	No	No
Private sector	Private veterinarians	Veterinarians working in a private capacity, e.g. ex-government veterinary officers or veterinarians working for NGOs	Yes	Yes
	Para-vets	Individuals who have received training from an NGO in livestock healthcare, usually around one month	No	No

### 3.2 Power dynamics operating in the value chain

#### 3.2.1 Institutional power (direct, collective)

Dallas et al. [47] describe institutional power as being most clearly exercised by the public sector via national regulation and legislation–i.e., for our study this includes the laws governing antibiotic production, distribution, and prescription. As ABR is a public health issue–an example of negative externality [57, 58]—governments and regulatory bodies are responsible in controlling value chain actors’ behaviours to minimise ABR. We therefore begin our analysis by understanding the extent to which sectoral regulations, guidelines, and access to infrastructure influence stakeholder behaviours.

*Lack of compliance with existing regulation*. To ensure appropriate antibiotic use by citizens, regulations exist in India governing how antibiotics move from pharmaceutical companies to end users. Wholesaler licencing stipulates antibiotics are only sold to businesses with a retail licence. Retail licence legislation states pharmacies employ a registered pharmacist to be present during business hours. Prescribing legislation limits the prescribing of antibiotics to veterinarians. In other words, regulations aim to ensure antibiotic delivery is overseen by experts with medical knowledge. However, these regulations were poorly enforced in our study sites and illegal distribution and prescription of antibiotics occurs. Medical representatives acting for pharmaceutical companies sometimes supplied antibiotics directly to livestock healthcare providers and livestock keepers, some livestock healthcare providers who did not hold retail licences obtained antibiotics directly from wholesalers, and qualified pharmacists were rarely present in pharmacies:

*“The reality is that hardly any time you will find them [pharmacists]*. *They will rather put their certificate on loan. So, if some moneyed man in village wants to start a chemist shop, he has to find a pharmacist in his locality, ‘why don’t you help me open my shop? I will give you 10,000 Rs or 20,000 Rs a month [approx. £100–200]’.” Pharmacology academics, Kolkata (KI-2-240919)*

Pharmacy and drug shop owners, not pharmacists, often dispensed antibiotics either over the counter or via an informal prescription written by livestock healthcare providers who lacked the legal mandate to do so, such as Pranibandhu and para-vets.

The Drugs Control Office in the Gram Panchayats, who were responsible for enforcing medicine governance, including regulating the area’s 3,000 to 4,000 pharmacies, employed just five staff to conduct inspections. This lack of public sector enforcement capacity was coupled with concerns of corruption that further limited the effectiveness of inspections:

*“If there is a raid and the drug inspector asks*, *‘where is your pharmacist?’ and the pharmacist is not there, which most of the time they are not, then the inspector can close the pharmacy. But the inspector will not, as immediately the inspector will be bribed. There is hardly any punishment in the public system. You can call it a kind of dishonesty or corruption.” Pharmacology academics, Kolkata (KI-2-240919)*

Consequently, commercially motivated actors were not obliged to conform to legislation designed to regulate, and potentially limit, their value chain activities.

*Lack of guidance regarding responsible and prudent antibiotic usage*. We also consider the role of professional associations such as veterinary statutory bodies and training centres in ensuring responsible and prudent antibiotic use. Here, another example of limited institutional power was the lack of antibiotic usage guidelines. We did not observe any government-based antibiotic usage guidelines and the only guidelines noted were those created and supplied by the veterinarians in Site 2 working for the NGO and in the government centres concerning production of small-scale commercial poultry systems. These written guidelines advised poultry producers to use antibiotics prophylactically to prevent respiratory and diarrhoeal diseases, including fluoroquinolones which are classified as highest priority critically important antibiotics for human health:

*“From our institution one chart has been prepared from day one to day 35 [of poultry production]*. *Say from zero to four days tetracycline is used and from 22 to25 days another antibiotic, say enrofloxacin, is used for all the [birds] […] If any problem arises in that period then they use other antibiotics, say tylosin for respiratory tract infection.” Veterinarian, Site 2 (KI-1-130120)*

The evidence from these findings suggests that existing regulations to ensure biomedical expertise regarding rational antibiotic use are not implemented and enforced. Moreover, the only antibiotic use guideline available was commercially motivated. Therefore, users lacked information that enabled them to use antibiotic appropriately. In fact, the converse was true, the information available to them and which they followed was sometimes inappropriate.

*The ‘veterinary service lacuna’ creates opportunity for informal providers*. In both study sites, livestock keepers only had access to a small number of veterinary medicine outlets–most of the pharmacies stocking veterinary medicines were in nearby towns rather than in the study sites. Furthermore, there was limited access to public veterinary services, with veterinary officers being stationed in local towns. This presented difficulties in accessing public livestock services, with livestock keepers citing the time and cost of getting to the block veterinary office as a barrier to access. Consequently, in both sites, livestock keepers received antibiotics through various livestock healthcare providers who lacked mandates to supply antibiotics to livestock, including human healthcare providers:

*“The [veterinary] doctor isn’t always available*. *But the people at the pharmacy are also doctors. We tell them I have a cow and it looks sick, give us some medicine.” Livestock keeper, Site 1 (LK-5-010719)*

While dual public-private practitioners—the Pranibandhu and Pranimitra- received no formal training on antibiotic use, para-vets working in site 2 received basic information on antibiotic use from the NGO. However, none of these stakeholders were legally allowed to prescribe antibiotics (Table 4). After training, Pranibandhu do not become officially employed by the government, working instead on a consultancy basis, being paid for the number of animals they artificially inseminate, a situation causing conflict among a group of Pranibandhu:

*“We appeal in the high court*. *We are here for so long, we must be given some permanent post. An order was given once [by the government] that we should be permanent. But final order is yet to be released, the dates are pending. We Pranibandhu, are under the government but we don’t get any salary from them.” Pranibandhu, Site 1 (SH-5-191119)*

Para-vets too, while retaining an association with the NGO training centre, receive no benefits from the government. The lack of legitimacy for these actors represents two problems. Firstly, livestock healthcare providers primarily generate income through medicine sales- including antibiotics- as described by this Pranibandhu:

*“The training we received as Pranibandhu has been on artificial insemination*. *We weren’t asked to prescribe medication*. *But if we did just that we won’t make enough money*, *so we*, *on our own*, *have learnt how to use antibiotics from other veterinary doctors*.*”* Pranibandhu, Site 1 (SH-5-110719)

Secondly, a void in prescribing control exists. The full gamut of prescribing habits cannot be monitored if the state refuses to acknowledge the role of informal providers in safeguarding animal health.

In summary, the lack of public and formal veterinarians encouraged informal livestock healthcare providers to emerge, most of whom prescribe antibiotics without the mandate to do so. As they are not recognised as formal antibiotic prescribers, they are unable to access formal training on appropriate antibiotic use and operate in a context where prescribing antibiotics is a tool to earn income and remains unmonitored by the state.

#### 3.2.2 Bargaining power (direct, dyadic)

Bargaining power is described as a coercive type of power, occurring when a dominant actor or firm can coerce others into doing something that they may otherwise not do [47].

*Pharmaceutical and wholesalers use incentives to promote antibiotic use*. Medical representatives working for pharmaceutical and wholesale companies can be considered to exercise bargaining power for their companies over other value chain stakeholders. Here, representatives use incentives, providing stockists and retailers with access to credit, discounts and gifts for bulk purchase, promotional and educational schemes, and their ability to be highly mobile within the physical space of the value chain, to influence purchasers to buy certain types and volumes of antibiotics. Furthermore, some medical representatives interact with livestock healthcare providers and livestock keepers directly to promote antibiotic use. One para-vet discussed his frequent interaction with medical representatives:

*“[I] have the phone number of medical representatives also*, *they sometimes come and tell [me] that this new medicine works better in this condition. And I used that [antibiotic] in the field, if it works I use it afterwards.” Para-vet, Site 2 (SH-1-130120)*

This again highlights the commercial incentives for antibiotic use. Medical representatives have opportunity to generate additional profit for their companies by promoting antibiotic use.

#### 3.2.3 Demonstrative power (indirect, dyadic)

When considering the type of demonstrative power operating in the value chain, we look to actors who are influencing others by establishing standards.

*Livestock healthcare providers mimic others prescribing habits*. We observed evidence of less experienced healthcare providers mimicking prescribing behaviour of more experienced providers–such as veterinarians–who exert demonstrative power by sharing their antibiotic practices within a local context:

*“We have learnt how to use antibiotics from other veterinary doctors*. *For example*, *we would accompany them during their visits and learn which antibiotics are used when*. *This is how we learnt it*.*” Pranibandhu*, *Site 1* (SH-5-110719)

Some of these experienced veterinarians operating in the study sites were seen to be promoting antibiotic stewardship practices, and attempting to influence other livestock healthcare providers:

*“We are trying to make the para-vets aware in a refresher training programme that you have to wait for three days*. *After [this], if it is not getting better then you can consult [us] and use antibiotics.” Veterinarian, Site 2 (KI-1-130120)*

Similarly, one pharmaceutical representative discussed how they use their interactions with healthcare providers to disseminate information on medicine usage:

*“We are doing a lot of para-vet conferences and company visits*. *We are trying to educate [them] in our own way. Some protocols are there, E. coli mastitis, different types of mastitis. We are giving information; which types of drugs are most effective.” Pharmaceutical representative, Kolkata (KI-%-200120)*

However, as reported earlier (Section 3.2.1), we observed evidence of veterinarians developing and using antibiotic guidelines which contain practices deemed inappropriate–namely the prophylactic use of fluoroquinolones in poultry. Consequently, transfer of antibiotic use experience from more to less experienced, and formal to informal, healthcare providers may not always align with ideal antibiotic stewardship goals.

These instances are examples of how livestock healthcare providers maintain a close relationship with other value chain actors within which information on antibiotic use is shared. While the relationships are not formalised, formal veterinarians and medical representatives express demonstrative power regarding common practices of antibiotic provision over other less experienced livestock healthcare providers.

#### 3.2.4 Constitutive power (indirect, collective)

Here we look to the social norms and understandings which surround antibiotic use and resistance in the study, to–as Dallas et al. [47] describe–“operate as a transmission mechanism for institutional reform”.

We observed varying levels of knowledge among our study participants concerning antibiotic use and resistance. Veterinarians and some medical representatives and livestock healthcare providers discussed concepts aligned with allopathic antibiotic stewardship such as spectrum of activity, diagnostic testing, and the negative consequences of antibiotic use such as side effects and resistance.

The responses of several livestock healthcare providers indicated that they had learned about antibiotics through many years of experience. Both the positive and negative effects of antibiotic usage were discussed, including possible side effects in livestock such as poor growth or abortion:

*“Once I used sulpha drugs [such as the antibiotic sulfadiazine] for diarrhoea but then abortion happened*. *So*, *I stopped using sulpha drug in diarrhoea cases*.*”* Para-vet, Site 2 (SH-1-130120)

Among pharmacy and drug shop owners and attendants, knowledge of antibiotics was variable, with some being unable to correctly identify antibiotics, reflecting the lack of formal training these stakeholders had received, as most are not pharmacists. Similarly, livestock keepers’ knowledge was also varied; while some were able to name certain antibiotics and demonstrate understanding of how antibiotics should be used properly—for example by following advice to complete a course of treatment—others thought that antibiotics could be used to treat viruses or were unfamiliar with the word ‘antibiotic’. Of those unfamiliar with the term antibiotic, further questioning revealed that many livestock keepers referred to the pharmaceutical treatments given to their livestock in broad terms such as ‘medicines’, ‘tablets’, or ‘injections’.

*Antibiotics are considered essential for treating a range of conditions*. What pervaded those stakeholders familiar with antibiotics was the close association antibiotics have with ‘modern’ medicine, with people talking about how antibiotics alleviate fear of bad outcomes and create confidence, and are therefore an integral part of healthcare:

*“If I am not using antibiotics in cows and goats*, *there is no confidence whether it will be cured or not*. *If I use antibiotic I am confident that the animal must get well*. *If I don’t use [antibiotics]*, *it would feel bad in my mind*.*” Para-vet*, *Site 2* (SH-1-130120)

“If a cow has a fever and I don’t use oxytetracycline, will that fever go down? I have to save [the cow]. Is there any way that without giving antibiotic the fever can be reduced? Is there any way the government of India [can] circulate [advice] among us?” Veterinary drug shop owner, Site 1 (SH-2-191119)

In addition, both livestock healthcare providers and livestock keepers have experienced that antibiotics are effective healthcare tools to prevent and treat illness in animals. These personal experiences have reinforced the notion that antibiotics are powerful, and therefore desirable, in treating disease and boosting farm productivity:

*“In poultry it is very much necessary to use antibiotics*, *in case of animal [livestock] it is ok [not to], but in poultry, without antibiotic it is very hard to stop mortality. It is like that antibiotic has to be used every day.” Para-vet, Site 2 (SH-2-130120)*

*“When symptoms like fever occur, and two or three days [have] passed but it does not go down, if you give antibiotics it is cured quickly.” Pranimitra*, *Site 2 (SH-3-150120)*

Consequently, social demand for antibiotics, whether appropriate or inappropriate, continues to be generated at the end of the value chain.

While some value chain actors demonstrated knowledge of antibiotic use in line with allopathic ideas of stewardship, many had limited knowledge, and commercial incentives to generate profit and obligational incentives to satisfy desires for positive clinical outcomes, may thus override opportunities to limit antibiotic use.

In summary, our power dynamic analysis demonstrates that the government currently lacks institutional power to influence value chain actors’ behaviours to use antibiotics appropriately. Pharmaceutical companies, wholesalers, and retailers motivated by profit generation face little interference from the state.

Because of the limited public sector capacity to provide adequate number of veterinarians for rural livestock keepers, for-profit actors have emerged at the end of the value chain to meet the demand for livestock healthcare, yet currently lack legitimacy and access to training opportunities to improve antibiotic use practices. Furthermore, a lack of guidelines for responsible and prudent use compounds the influence of for-profit actors and prevents deviation from strong social norms which continue to associate antibiotics as the most efficacious and safe therapeutic option for many diseases. As such, commercial interests and pressure to prescribe may incentivise stakeholders to deviate from appropriate use.

## 4. Discussion of implications for antibiotic stewardship

In this section, we discuss how the structure of the antibiotic value chain and the power relationships therein have implications for antibiotic stewardship in the study sites, with a summary provided in Table 5.

**Table 5:** Power dynamics operating in the antibiotic value chain and their implications for stewardship.

Power type (and dimensions of power) [47]	Study finding	Considerations for antibiotic stewardship (authors’ opinions)
***Institutional*** *(direct and collective)*	Limited enforcement and implementation of existing regulations regarding antibiotic distribution and dispensing	Antibiotic stewardship needs to look at why a lack of enforcement capacity exists and consider whether making changes to this level of governance could improve antibiotic use
	Lack of antibiotic usage guidelines for therapeutic use in animals	Independently produced, evidence-based treatment guidelines should be developed in line with international standards
	Limited public sector capacity to provide access to sufficient, good quality veterinary services to rural populations	Stewardship efforts need to address the structural deficiencies in the system by 1) increasing the number of formally trained professionals in the system, 2) harnessing the relationships between veterinarians and other livestock healthcare providers to amplify veterinary capacity, and 3) increasing the number of veterinary drug outlets, for example by adding veterinary counters in human drug shops
	Ambiguous status of informal veterinary healthcare providers. Informal providers overstep their role by supplying antibiotics to livestock	Improving the legitimacy of the livestock healthcare providers may increase their access to adequate training on responsible use of antibiotics and help to improve their prescribing habits
***Bargaining*** *(direct and dyadic)*	Pharmaceutical and wholesaler’s use of incentives to promote antibiotic use and profit commercially	Guidance for antibiotic use may be motivated for profit and not concerns for public health. Stewardship which threatens livelihoods is likely to face challenges and so research is needed to understand how business models could align with stewardship principles
***Demonstrative*** *(diffuse and dyadic)*	Informal healthcare providers learn from more experienced value chain actors	Influential stakeholders could be utilised to promote antibiotic stewardship, e.g. through mentoring and leading by example
		Inexperienced stakeholders require access to antibiotic training.
***Constitutive*** *(diffuse and collective)*	Culture of misconceptions that without antibiotics you cannot treat disease	Confidence in alternative treatment options needs to be bolstered through the dissemination of information and training

Our research shows that access to livestock healthcare and antibiotics exists within a pluralistic veterinary health system where public and private, and formal and informal antibiotic providers operate. This is similar to the provision of healthcare and antibiotics in the human health sector in India [59] and has also been reported by Arnold et al. [18]. The presence of numerous informal antibiotic providers is likely to be a result of a deficiency of the state to provide access to veterinarians in rural locations as it is currently estimated that India has access to only half of its required veterinarians [25].

Consequently, numerous actors seek opportunity to earn a living by filling this ‘veterinary service lacuna’. While many of the livestock healthcare providers are not legally allowed to prescribe antibiotics, the finding that this practice was widespread was consistent with other LMICs [60], and has been reported in different livestock [20, 21, 24] and human [61] settings in India. In study site 2, where an NGO was in operation, there did appear to be a system of providing continued training on antibiotics for the para-vets associated with the NGO—which is encouraging. Study site 2 has been the beneficiary of several area development programmes for agriculture which have not occurred in site 1, which may be due the latter being less rural and closer to Kolkata.

ABR is a complex negative externality issue, affecting the lives and livelihoods of users themselves and others, and occurring with a time-lag: actions today do not have an immediate consequence. Thus, markets alone cannot incentivise value chain actors to control ABR. Consequently, the state and citizens must intervene. Public bodies—governments and regulators–need to be responsible for shaping value chain actors’ behaviours through regulations, enforcement and incentives so that they use antibiotics appropriately. However, citizen intervention is challenging when awareness and knowledge on the issue is lacking and people have commercial, livelihood, and/or health interests that go against antibiotic stewardship.

Our research shows that the government’s influence–i.e., their institutional power–is lacking in three key aspects. Firstly, existing regulations regarding the licencing of antibiotic distributors (wholesalers and retailers) and qualification of antibiotic prescribers are not effectively enforced because of staff shortages, stakeholder’s vested interests, and the discrepancy between regulatory requirements and reality of limited access to qualified prescribers. For example, while retail legislation stipulated that pharmacies were operated by people with pharmacology training, this was rarely the case and consequently antibiotics were dispensed by non-pharmacists. Kotwani et al. [62] report similar findings in their study where only one of the four wholesalers interviewed in Delhi had a pharmacy degree and participants raised concerns over the shortage of public sector staff to conduct inspections.

Secondly, we observed no proper guidelines for responsible and prudent use in the study sites. As a result, guidelines developed for profit driven actors such as those for commercial poultry production in Site 2 include the routine use of fluoroquinolone antibiotics for prophylaxis. This type of practice is deemed inappropriate by the Food and Agriculture Organization of the United Nations, the World Organisation for Animal Health, and the Indian National Action Plan on ABR [7, 63, 64]. Indeed, these organisations have produced manuals and strategies at the international and national levels which could be used to guide stewardship efforts [7, 63]. However, these have yet to be adapted to the Indian context and made available to stakeholders in our study sites. Consequently, we observed numerous instances of antibiotics belonging to classes deemed critically important for human health, including antibiotics packaged for human use, being used in livestock [18].

Thirdly, the public sector could not meet the demand for veterinary healthcare, which led to for-profit actors and informal healthcare professionals, both livestock and human, prescribing antibiotics to livestock. These actors, operating in an institutional power void, exercise their bargaining power to generate profit from antibiotic sales. For instance, pharmaceutical and wholesale companies employed representatives to conduct marketing for their products. While further research is needed to assess how profit margins vary across different antibiotics, and how this may influence prescribing decisions, Nguyen [65] highlights that the commercial interests by private healthcare providers can lead to prescription of more drugs than public providers. In the human medical sector in India, pharmaceutical representatives engaging in clinical training of doctors attempted to influence prescribing in favour of the representatives’ company [66, 67]. Indeed, these actions of actors operating towards the end of the antibiotic value chain benefit those higher up, such as pharmaceutical companies, by generating sales.

While we cannot assume that informal providers’ practices are worse than formal providers, their lack of formal training may increase the likelihood of this happening as inexperienced stakeholders were reliant on mimicking the prescribing patterns of other antibiotic users, an example of demonstrative power operating in the value chain. Indeed, there was substantial variation in the level of antibiotic training received by prescribers as discussed. Even when expertise was available through trained pharmacists, they were mostly absent from drug shops and therefore their knowledge was not transferred to antibiotic prescribers and users. This indicates the need to target antibiotic stewardship interventions to all prescribers–both formal and informal–as suggested by Gautham et al. [59].

In our study sites antibiotics remain closely entangled with concepts of ‘modern’ medicine and continue to be used as first line treatment, often in cases where this may not be clinically necessary. This social norm of antibiotics being essential for the treatment of disease–an example of constitutive power operating in the value chain—has been reported by others: Pearson and Chandler [8] describe antibiotics as care and hygiene and Tompson and Chandler [68] discuss how antibiotics are ‘quick fixes’ and argue that to move away from this status quo interventions need to address the societal structures within which antibiotics exist.

Our power analysis demonstrates that work is needed on multiple fronts of the power framework: not just strengthening institutional power, but also building constitutive and demonstrative power. Based on this evidence, our recommendation to improve antibiotic stewardship in West Bengal is twofold and looks beyond simply suggesting government increase their enforcement capacity, an action which could deliver real change but is likely to face significant economic barriers. Furthermore, given the lack of veterinary infrastructure, implementation and enforcement of existing antibiotic legislation could leave many livestock keepers without access to treatments, with negative consequences to livelihoods and animal health and welfare. Moreover, veterinarians too may have knowledge limitations which need to be addressed in their pre-service training.

First, the public sector could develop treatment guidelines for the animal sector which are aligned with antibiotic stewardship goals and international standards (e.g., the World Organisation for Animal Health’s terrestrial animal health codes and list of antibiotics of veterinary importance). For livestock healthcare providers, deferring to alternative treatments or pricing structures which prioritise payment for services may alleviate this pressure. However, this may be more challenging when considering adoption of stewardship by pharmaceutical companies and medical representatives whose business models are focused on sales volumes. For commercial livestock, improvements in biosecurity and animal husbandry practices are needed to support production so that enterprises are not left vulnerable without antibiotics.

Second, the dissemination of newly-developed guidelines and provision of training opportunities needs to include all veterinary healthcare providers–including informal antibiotic providers, thus urging the state to recognise their value as essential animal health care providers. However, providing legitimacy requires careful considerations and consultation with different stakeholders. In their work with the World Organisation for Animal Health, Cobbold et al., [69] describe the collaborative methods needed between individuals and agencies to understand the role veterinary paraprofessionals take in service delivery. This work produced a set of competency guidelines which provide information on the knowledge and skills expected of veterinary paraprofessionals after effective training. The authors argue that these guidelines can be used to assist countries in training paraprofessionals and serve as a catalyst in the review of regularity standards.

Currently, literature linking value chain and power analysis to the development of effective interventions in livestock and healthcare sectors is lacking. In their report on ‘value chain development for decent work’, the International Labour Office set out a framework for value chain development [70], which includes steps on intervention design, implementation, and monitoring results. Here, the authors use a case study of how value chain analysis has been used to design interventions to improve employment opportunity for Serbians with vocational training. Consequently, efforts should be taken to carefully document the implementation of any antibiotic stewardship interventions based on our study to strengthen the case for future value chain and power dynamic analysis for related healthcare and livestock challenges.

Using a framework for value chain power development set out in Dallas et al., [47] we hypothesise that our suggested interventions could provide the means for public and private stakeholders to improve antibiotic usage, deliver effective training, and include all livestock healthcare providers in their monitoring and inspection procedures. This could help to address the veterinary service lacuna by providing a more formal and regulated cadre of antibiotic providers. An increase in institutional power of this type should contribute to the constitutive power operating in the chain by making knowledge of good practices common across all antibiotic user groups and thereby a widely accepted norm. Over time this may alleviate some of the social norms of antibiotics acting as panaceas, especially if alternatives are more commonly used, and thus reduce antibiotic demand by end users. Subsequently, the use of good antibiotic practices by early adopters may encourage others to do the same, an example of building demonstrative power.

Limitations exist within this study. Qualitative research data may be biased because of hidden agendas, assumptions, and the etic lens of the research team; as researchers working on antibiotic use and stewardship we are biased towards the view that antibiotic reform is a worthwhile endeavour and in the public interest. Social desirability bias may have been introduced by those participants aware that their antibiotic practices may be inappropriate. Furthermore, the construction of ‘truth’ within our findings occurred through interactions between ourselves, translators, and those being interviewed i.e., a critical realist ontological position and it is possible that alternative ‘truths’ may exist. For example, basing our household selection criteria on livestock type and production system may have overlooked differences introduced by variation in participant gender, education level, and socioeconomic status. However, we sought to limit these potential issues by using non-leading open ended questions [44], gathering information from a range of sources, and by working with local partners embedded to the research setting who could provide a more emic view, i.e., attempting to triangulate our research findings.

During the study we also sought to question stakeholders about antibiotic quality. However, these questions proved challenging and the answers did not provide robust information to include in our analysis. Additionally, we anticipated discussion of antibiotic use would reveal more information regarding the social value of antibiotics to those using them. It is possible that this line of enquiry was hindered due to many of our questions focusing specifically on antibiotics rather than medicines in general, and the fact that many livestock keepers were not familiar with the term antibiotics. Consequently, additional study could involve detailed ethnographic work focusing on how interactions with medicines shape livestock keepers’ experiences of interacting with healthcare. Despite these limitations, our study provides a granular account of antibiotic distribution across the study sites and the power dynamics operating therein which could impact the development of antibiotic stewardship interventions.

Further work to investigate antibiotic value chains could include additional exploration of how stakeholders interact with and consider substandard and falsified medicines, as this issue was outside the scope of our project but has been identified as a topic of interest in antibiotic stewardship, particularly in Asia, Africa, and Latin America [71, 72]. Quantitative research could be undertaken to assess the volumes and type of antibiotics being used at farm/animal level, particularly to compare the public and private sector distribution, and to understand how quality and formality of antibiotic use differs across livestock sub-sectors. Furthermore, research could investigate the role of antibiotic profit generation in influencing prescribing practices in the private sector, and how this is affected by antibiotic procurement challenges faced by the public sector.

## 5. Conclusions

This paper describes how antibiotic distribution to livestock in rural West Bengal occurs through numerous routes and nodes, both formal and informal, many of which circumvent legislation designed to control how antibiotics are governed. Given the current lack of enforcement capacity governing antibiotic distribution and the frequency of informal antibiotic prescribing that occurs in livestock healthcare, it is unlikely that attempts to mandate antibiotic prescribing alone will be viable stewardship options. Alternative options could include addressing public sector deficits, with respect to both service and antibiotic provision and by providing resources such as independent and locally relevant antibiotic guidelines. When disseminating these guidelines, all antibiotic prescribers—both formal and informal–should be included to increase the legitimacy of all livestock healthcare providers and the quality of antibiotic use in the value chain.

By describing and analysing the antibiotic value chain and considering the various power dynamics operating therein, we identified several possible entry points for antibiotic stewardship. These entry points will need to be investigated in further detail, in consultation with value chain stakeholders, to ascertain the potential for related interventions to improve the quality of antibiotic use in the two study sites we investigated and in other similar resource scarce settings.

## Supporting information

S1 File Key informant interview guide. (DOCX)

S2 File Stakeholder interview guide. (DOCX)

S3 File Household interview guide. (DOCX)

## List of abbreviations

ABR: Antibiotic resistance
KI: Key informant
LMICs: Low- and middle-income countries
LK: Livestock keeper
LS: Livestock
MRs: Medical representatives
NGO: Non-governmental organisation
SH: Stakeholder
VO: Veterinary officer
