# Supplementary material for: Pharma-cartography: Navigating the complexities of antibiotic supply to rural livestock in West Bengal, India, through value chain and power dynamic analysis
Source: PLoS One. 2023 Feb 2;18(2):e0281188. doi: 10.1371/journal.pone.0281188 (PMC9894437; doi:10.1371/journal.pone.0281188)
Supplement: S1 File — (DOCX) [file pone.0281188.s001.docx]

**A multi-stakeholder approach towards operationalising antibiotic stewardship in India’s pluralistic rural health system.**

**Mapping of supply and value chain**

**DRAFT Key Informant Interview Guide - Antibiotic supply chain stakeholders**

**Objective:** To gain a better understanding of:

- *Value chain system structure for human and animal antibiotics (AB), which includes antibiotics flows, people or institutions involved and spatial locations of nodes.*
- *Assess key governance factors influencing the chain, including identification of regulators, decision-makers, power groups and formal and informal norms*
- *Nature of barrier(s) and incentive(s) involved in the antibiotic supply chain.*
- *Identification of overlaps of ABs between human and animals, and the reasons for this overlap.*

**Potential participants:** The interviews will involve people with high level of knowledge of some of the supply chain system (e.g. pharmaceutical companies representatives, medical or veterinary associations, etc.).

Two type of interviews will be conducted:

- Interviews that aim at mapping the chain (PARTICIPANT TYPE 1)
- Interviews that aim at validating and understanding interactions (e.g. regulations) in the chain (PARTICIPANT TYPE 2)

**Guidance for interviewers and note-takers**

**Introduction**

Before investigating the key topics of investigation, the researcher will provide an information sheet and discuss the project overview, the interviewer background, purpose and duration of the interview and provide the opportunity for questions. The researcher will then present the consent form for signing by the participant. The interview will proceed only once the consent form has been signed. Once the consent form is signed, the voice recorded should be activated.

**Before the interview, please ensure the following**

- You have gone through the interview guide and have familiarised yourself with the questions
- The recording device is working and is set up properly
- You have enough pages in your notebook to note the conversation, in case the provider does not consent to being recorded.

**At the beginning of the interview**

- Explain/read out all the required information about the research and the confidentiality issues before starting with the questions. Please use the information provided in the informed consent form.

**During the Interview**

- Try not to refer to the guide during the interview, but at the end make sure you have covered all the topics. Do not read out the questions.
- Ask the questions in a logical manner, and not necessarily in the same order as the topic guide. If the interviewee has already fully answered a particular question spontaneously do not ask the same question again just because it is the next question in the topic guide.
- Do not ask very sensitive questions in the beginning as this will make the stakeholder suspicious. For example, do not ask questions about incentives from antibiotics at the beginning.
- Remain attentive and listen carefully to each answer. Do not interrupt when the interviewee is speaking unless the discussion is going into a totally irrelevant area.
- Before asking any question, think about how you are wording the question. Please do not ask leading questions. Keep them open ended and do not give the answer in your question. For example, instead of asking poultry farmers early on in the interview ‘Do you mix antibiotics in your chicken feed?’, ask ‘What are the ingredients that you mix in the feed for your chicken?’

**At the end of the interview**

- Make sure you obtained the interviewee’s signature on the consent form.
- Try to get some pictures, especially if the setting is interesting.
- If the pictures include any clearly visible and identifiable human subjects, it would be best to get their signed consent as this is required for any kind of publishing of photographs.

**Key Topic 1: Role of stakeholders in the value chain.** [ASK TO ALL PARTICIPANTS]

Background of the stakeholders-

- *Please describe your profession and the community you serve*
  - ***Prompt:*** *probe for sectors, groups, segment of individuals.*
- *How long have you been working in this profession*
  - ***Prompt:*** *geographical area (*national/ regional/ local level)*?*
- *Can you tell me how you started working in this profession?*
  - ***Prompt:*** *Why did you decide to take this career path?*

**Key Topic 2a: Mapping of the AB value chain** [ASK TO PARTICIPANTS TYPE 1]

INTERVIEWER TO DRAW A MAP OF THE VALUE CHAIN TOGETHER WITH THE KEY INFORMANTS, USING A BLANK SHEET OF PAPER. THIS IS AN INTERACTIVE PROCESS AND BOTH THE INTERVIEWER AND THE PARTICIPANTS NEED TO AGREE ON WHAT SHOULD BE WRITTEN IN THE DIAGRAM.

*We would like you to help us draw a diagram on how the antibiotic supply system works for people or for animals in rural areas of X (district) [ FOR THIS CAN BE REGIONAL RURAL LEVEL, BUT FOR SOME INFORMANTS UPSTREAM CHAIN NODES LOCATED AT NATIONAL LEVEL -PROBE] . For this:*

- *Can you please describe or draw a diagram on how antibiotics reach households [or livestock holders]?*

*The interviewer to probe (the order of probes depends on the type and knowledge of the key informant):*

- - *Differentiate type of antibiotic final users:*
    - *Human AB use: Households, clinical centres (local health centres, hospitals- public and private), and any other place where antibiotics are being administered to people (pharmacies, drug shops, other)*
    - *Livestock AB use: Type of livestock production systems (commercial or backyard) and species kept (poultry, dairy, beef, sheep, goat, poultry, pig, other important systems)*
  - *Differentiate type of providers of antibiotics to final users (e.g. type of providers such as doctors, health technicians, drug shop sellers, etc)? Example of questions:*
    - *How would you differentiate the type of ‘X’ (e.g. provider) operating in the region. [provide freedom for the key informant to make his own classification]*
      - *Probe: Differentiation based on:*
        - *method of operation,*
        - *size of operation*
        - *type of training or experience*
        - *type of clients used.*
      - *Ensure that the definition or characteristics of each provider are well understood. For example, if herbalist, please ask them to describe what type of stakeholder is this and how are these different from other providers.*
  - *Repeat the previous question with [ENSURE CAPTURE THOSE KNOWLEAGEABLE TO THE KEY INFORMANT]:*
    - *Pharmacists*
    - *Wholesalers, stockists, sub-stockists and local stockists*
    - *Clearing and forwarding agents*
    - *Producers or pharmaceutical companies*
    - *Formal doctors (if not mentioned before)*
    - *Informal providers to people (if not mentioned before)*
    - *Animal health providers (if not mentioned before) (ensure you differentiate all the different types: parabendus, herbalists, private vet etc..)*
    - *Formal veterinarians (if not mentioned before)*
    - *Livestock enterprises*
    - *Animal feed manufactures*
    - *Antibiotic disposal agencies*
    - *Any other node identified (e.g. clinical centres, etc.)*
- *Work out contribution of price along the chain:*
  - *If consumer buy an antibiotic at 100 ruppes, what would be the price of buying of this antibiotic by:*
    - *Each provider (doctor, stockist, pharmacist, etc) – Use the two main chains to assess this. [USE THE SAME APPROACH AS WITH THE PILOT INTERVIEW]*
- *What happens to the antibiotics after the end of their shelf life?*
  - ***Prompt:*** *Who deals with them? And how?*
  - ***Prompt:*** *Can these be used by providers and consumers through informal chains? If so, please describe how these chain operates*

[THE DIAGRAM SHOULD INDICATE WHO DEALS WITH ANIMALS AND HUMAN ANTIBIOTICS]

- - *For each people identified, probe:*
    - *To who do they sell or administer antibiotics?*
    - *From whom do they buy or get their antibiotics? [ THIS IS TO ENSURE ARROWS ARE DRAWN IN THE DIAGRAM TO CAPTURE FLOWS AND CONNECTIONS BETWEEN VALUE CHAIN STAKEHOLDERS]*
  - *Looking at the diagram, what are the most common flows of antibiotics?*
    - *Why?*
    - *Interviewer to explore possible proportion of flows? (e.g. what proportion of households use informal providers? Or what proportion of informal providers get their AB supply from X?) if not possible, ask the participants to identify for each end users (type of households or livestock system) those chains that are:*
      - *Very frequently used*
      - *Commonly used*
      - *Rarely used*
      - *Very rarely used*
    - *Why?*

*[Quantification needs to be done separately for animal and human antibiotic usage]*

**Key Topic 2b: Validation of the map of the AB value chain** [ASK TO PARTICIPANTS TYPE 2]

*INTERVIEWER TO SHOW AND EXPLAIN THE MAP OF THE CHAIN OBTAINED, AND THEN ASK:*

- *Do you agree with this representation of the chain?*
  - *Prompt: Why / Why not?*
  - *Prompt: Please explain what would you change this diagram? why?*
- *Are there any important types of people or node missing in this diagram?*
  - *If yes, which one? How would you incorporate it? What is their role in the system?*
  - *If yes, how would you differentiate these type of people?*
    - - *Probe: Differentiation based on:*
        - *method of operation,*
        - *size of operation*
        - *type of training or experience*
        - *type of clients served.*

**Key Topic 3: Quality of AB and their flows** [ASK TO PARTICIPANTS TYPE 1]

- How do you differentiate the different quality of antibiotics in the system?
- ***Prompt:*** *What would make the antibiotic to be in the best quality category? Why?*
- ***Prompt:*** *What would make the antibiotic to be in the lower quality category? Why?*
- In the diagram, could you indicate who deals with the different type of quality of antibiotics? [INTERVIEWER TO MARK IN THE VALUE CHAIN DIAGRAM WHO DEALS WITH EACH TYPE OF ANTIBIOTICS]
  - **Prompt:** Why or what are the factors for a given stakeholder to use low quality antibiotics?
  - **Prompt:** Why or what are the factors for a given stakeholder to use good quality antibiotics?
  - **Prompt:** What, in your opinion, are the consequences of using low quality antibiotics?
  - **Prompt:** If more than one quality use by a stakeholder, please explain why would this be the case?

**Key Topic 4: Governance and methods of operation** [ASK TO ALL PARTICIPANTS – FOR PARTICIPANT 2 IT CAN BE TARGETTED TO SPECIFIC SECTIONS OF THE CHAIN]

- What are the main reasons for ‘X’ (e.g. backyard livestock owner) to use ‘Y’ (e.g. informal medical provider)? THIS CAN BE DONE WHILE DRAWING THE CHAIN DIAGRAM. FOR EXAMPLE, IF HERBALIST IS IDENTIFIED, THE RESEARCGER TO ASK WHY DO THEY USE THESE INSTEAD OF OTHER Y.
- Explain how ‘X’ (e.g. informal provider) people operate to administer or sell antibiotics? THIS IS ABOUT INCENTIVES.
  - What are the incentives used by X to sell antibiotic to Y? why? (or how do X convince Y to buy their antibiotics?) What is their normal strategies to sell or administer antibiotics? Why?
  - How do they influence their clients to get their antibiotics from them?
  - How do they influence their supplier to sell them antibiotics?
  - **Prompt:** what type of credit arrangements exist between suppliers and providers, and providers and users?
  - What is their strategy for storing and resupplying of antibiotics?
  - How do they deal with antibiotics that have passed their sell by date (expired drugs)?

REPEAT THE PREVIOUS SET OF QUESTION FOR EACH STAKEHOLDER

- To your knowledge, who are the most influential people in the system for the selling or usage of antibiotics? Why?
  - **Prompt:** are there any major associations that have a major influence on these people at the rural level?
- How people get training on antibiotic usage across the chain [ask for different people]?
  - **Prompt:** who are the people providing the training?
  - **Prompt:** are there any official guidelines available? By which stakeholders? To what extent? Who produces the guidelines?
- How are new antibiotics introduced into the market?
  - **Prompt:** who the people who come to talk about new products?
  - **Prompt:** are samples of antibiotics or promotion materials provided?
- Can you indicate who are the people or institutions regulating the stakeholders?
  - Prompt: What role do they have?
  - Prompt: how is enforcement performed? At what level (local, regional or national level)?
  - Prompt: What is the impact of the enforcement? How effective is it? Why? What are the main barriers for enforcing the regulations? Whom does this affects the most? Why?
- In your view, what are the main barriers for adequate use of antibiotic in the system?
  - Prompt: Why? (probe for patient expectations, cost, lack of enforcement, conflict of interests of drug suppliers/ prescribers, risk of food insecurity etc)
  - Prompt: What are the aspirations for better use of antibiotics?
  - Prompt: How can this be achieved? What are the main barriers to achieving this?
- In your view, what type of interventions do you think would be useful/effective to control or improve the use of antibiotics in the system?
  - Prompt: what would be a useful stewardship intervention? Why?

**Key Topic 5: Investigation of potential overlaps of human and animal antibiotic usage at the household and farm level [**ASK TO ALL PARTICIPANTS]

- *Are there any situations where human antibiotics are used in animals?*
  - *Prompt: Where in the chain (and production systems) is it more likely to occur?*
  - *Prompt: What are the main reasons for this?*
- Are there any situations where people in the human antibiotic sector influence or provide advice for antibiotic usage in animals?
  - *Prompt: Where in the chain is it more likely occur?*
  - *Prompt: What are the main reasons for this?*
- *Are there any situations where animal antibiotics are used in humans?*
  - *Prompt: Where in the chain is it more likely occur?*
  - *Prompt: What are the main reasons for this?*
- Apart for the situations mentioned, are there any situations where people in the animal antibiotic sector influence or provide advice for antibiotic usage in humans?
  - *Prompt: Where in the chain is it more likely occur?*
  - *Prompt: What are the main reasons for this?*

**THANK YOU**
