# Supplementary material for: Pharma-cartography: Navigating the complexities of antibiotic supply to rural livestock in West Bengal, India, through value chain and power dynamic analysis
Source: PLoS One. 2023 Feb 2;18(2):e0281188. doi: 10.1371/journal.pone.0281188 (PMC9894437; doi:10.1371/journal.pone.0281188)
Supplement: S2 File — (DOCX) [file pone.0281188.s002.docx]

**A multi-stakeholder approach towards operationalising antibiotic stewardship in India’s pluralistic rural health system.**

**Mapping of supply and value chain**

**DRAFT Stakeholder Interview Guide - Antibiotic supply chain stakeholders**

This guide will be used as a template for the semi-structured, qualitative interviews of various stakeholders involved in antibiotic supply chain in/close to the site of investigation.

**Objective:** We aim to gain a better understanding of:

- Current norms of antibiotic (AB) supply and role of the individual stakeholder in the supply chain.
- Nature of barrier(s) and incentive(s) involved in AB supply chain.
- Power groups (both government & non-government) involved and the nature of their influence.
- Identification of AB supply chains for human and animal use
- Identification of overlaps of AB between human and animals, and the reasons for this overlap.
- Perception on barriers and interventions needed

Potential participants:

The interviews will involve stakeholders who stock, supply and dispense the antibiotics (Holders –H) and stakeholders that influence the process (Influencers –I).

**Guidance for interviewers and note-takers**

**Introduction**

Before investigating the key topics of investigation, the researcher will provide an information sheet and discuss the project overview, the interviewer background, purpose and duration of the interview and provide the opportunity for questions. The researcher will then present the consent form for signing by the participant. The interview will proceed only once the consent form has been signed. Once the consent form is signed, the voice recorded should be activated.

**Before the interview, please ensure the following**

- You have gone through the interview guide and have familiarised yourself with the questions
- The recording device is working and is set up properly
- You have enough pages in your notebook to note the conversation, in case the provider does not consent to being recorded.

**At the beginning of the interview**

- Explain/read out all the required information about the research and the confidentiality issues before starting with the questions. Please use the information provided in the informed consent form.

**During the Interview**

- Try not to refer to the guide during the interview, but at the end make sure you have covered all the topics. Do not read out the questions.
- Ask the questions in a logical manner, and not necessarily in the same order as the topic guide. If the interviewee has already fully answered a particular question spontaneously do not ask the same question again just because it is the next question in the topic guide.
- Do not ask very sensitive questions in the beginning as this will make the stakeholder suspicious. For example, do not ask questions about incentives from antibiotics at the beginning.
- Remain attentive and listen carefully to each answer. Do not interrupt when the interviewee is speaking unless the discussion is going into a totally irrelevant area.
- Before asking any question, think about how you are wording the question. Please do not ask leading questions. Keep them open ended and do not give the answer in your question. For example, instead of asking poultry farmers early on in the interview ‘Do you mix antibiotics in your chicken feed?’, ask ‘What are the ingredients that you mix in the feed for your chicken?’

**At the end of the interview**

- Make sure you obtained the interviewee’s signature on the consent form.
- Try to get some pictures, especially if the setting is interesting.
- If the pictures include any clearly visible and identifiable human subjects, it would be best to get their signed consent as this is required for any kind of publishing of photographs.

**Key Topic 1: Role of stakeholders in the value chain.**

Background of the stakeholders- [ASK TO ALL PARTICIPANTS]

- *Please describe your profession and the community you serve*
  - ***Prompt:*** *probe for sectors, groups, segment of individuals.*
- *How long have you been working in this profession*
  - ***Prompt:*** *geographical area?*
- *Can you tell me how you started working in this profession?*
  - ***Prompt:*** *Why did you decide to take this career path?*

**Key Topic 2: Mapping the value chain and factors motivating stakeholder choice – What factors decide stakeholder participation in value chain?**

**Antibiotics used:**

- **What are the antibiotics you deal with?**
  - **Prompt:** Ask the stakeholder to show you the antibiotics stocked or the list of antibiotics they work with. If they do not know what an antibiotic is, please ask to see the medicines used (or the list of medicines) in his business. [if possible take a picture or record the type of antibiotic used]
  - **Prompt:** what are the most frequent antibiotics use/sold in your business? Why?
    - **Prompt:** for livestock?
    - **Prompt:** for humans?
    - **Note:** if too many, please ask the first three for humans and livestock
    - ***Prompt:*** *Estimate volume share of antibiotics in your business? (proportion of drugs sold that are antibiotics?)*
    - ***Prompt:*** *What proportion of your profits depend on antibiotics selling?*
    - ***Prompt:*** *Within antibiotics, which AB classes have the bigger shares at present?*

The next questions should be done for the different type of antibiotics identified

Supplier/ upstream factors. [FOR ALL PARTICIPANTS EXCEPT PRIMARY PRODUCERS OF AB, ALTHOUGH NEED TO PROMPT IF THEY BUY ANTIBIOTICS FROM OTHER SOURCES]

- *How are antibiotics supplied to your enterprise in the area?*
  - ***Prompt:*** *How many suppliers do you use?*
  - ***Prompt****: Can you describe the different type of suppliers you use? [Interviewer to write down the type of suppliers of AB]* *MAKE SURE THAT EAH SUPPLIER IS WELL DIFFERENTIATED. FOR EXAMPLE, THERE COULD BE MANY DIFFERENT TYPE OF STOCKISTS, VETERINARIANS, ANIMAL HEALTH PROVDERS, ETC. PROMPT CAN BE USED TO ASK FOR DIFFERENCES:*
    - ***What are the different type of X (e.g. stockist) you work with? How would you differentiate them? (if needed, ask for differences: e.g. what is the difference between an herbalist and a parabendu?)***
  - *What criteria do you use to select your antibiotic suppliers?*
  - *Who is your main supplier? Why?*
    - ***Prompt:*** *Can you explain how you ended up using them? How did you find them?*
    - ***Prompt:*** *How did they convince you to choose them as a supplier?*
    - ***Prompt:*** *What sort of arrangement do you have with this supplier for antibiotics?*
      - *Probe for offers, promotions, credit service, other incentives provided by suppliers…*
      - *Probe how are the different incentives work*
      - *Probe for contracts*
    - ***Prompt:*** *How do you get a resupply from them? How often? How does it work?*
    - ***Prompt*** *Does this supplier give you any information or advice on how to sell or use antibiotics? If yes, can you tell me what advice do they give you?*

[REPEAT THE LAST SET OF QUESTIONS FOR EACH PROVIDER IDENTIFIED]

- - *Have you changed any supplier of antibiotics in the past year?*
    - ***Prompt:*** *What was the reason for your decision?*
  - *Are there other supplier you would want to use?*
    - *If so, why are you not using them? What are the main barriers?*

Client-specific factors.

- - Who are the type of clients you serve? [If animal owner, then replace clients for type of animals]
    - ***Prompt:*** *Private individuals or households; Institutions,* *informal doctors, formal doctors, animal health providers, livestock owners, etc…* If animal owners, which type (poultry, cattle, dairy, sheep, goat, pig, horse)? [Interviewer to draw the clients]
  - *Of these, who are the main client?*
    - ***Prompt:*** *What kind of requests/ demand do this client generally have?*
      - ***Prompt:*** *Can you describe the common situation on how the process work?*
      - ***Prompt:*** *probe for clinical outcome, cost, quality of drugs/ do they ask for antibiotics directly? If so, why?…)*
    - ***Prompt:*** *What are the most common reasons for providing antibiotics?*
      - ***Prompt:*** *to people?*
      - ***Prompt:*** *to animals? [ differentiate poultry, cattle, sheep/goat, pigs, other]*
      - ***Prompt:*** *if animals, for feed or growth promoters?*
      - ***Prompt:*** *Who administers the antibiotics in animals? Why?*
    - ***Prompt:*** *Why do they come to you? What incentives do you offer to them to buy from you?*
    - ***Prompt:*** *How do these clients usually pay for their medication?*
      - ***Prompt:*** *Are they usually able to afford their medication? If not, do you allow a credit system for your client? What is the average period of credit?)*
    - ***Prompt:*** *What are the main difficulties you experience with these clients when giving antibiotics? [if lack of prescription is an issue, ask what happens]*
    - ***Prompt:*** *Do they come to you for advice on antibiotics? Why? In which situations?*
    - ***Prompt:*** *Do you give them any advice on how to use antibiotics? Why? What type of advice?*

[REPEAT THE LAST SET OF QUESTIONS FOR EACH CLIENT IDENTIFIED]

- - ***Prompt:*** do you serve other communities too? How far? For which type of clients?

Competition specific factors.

- - *Are their similar providers of antibiotic in your area?*
  - ***Prompt****: if yes, do you interact on a regular basis? How? Do you share information/ knowledge?*
    - ***Prompt:*** *Is there a difference between the clients both of you serve?*
  - *What are the differences between you and these other providers? Why?*
  - *Are these other businesses affecting the way you give antibiotics?*
    - ***Prompt:*** *How?*
    - ***Prompt:*** *How else are these affecting you?*

**Topic 3. Influence and governance: Who are the people who affect your decision making when purchasing or supplying antibiotics?**

Factors related to influencers

- - *Where do* you get information *about antibiotics?*
    - ***Prompt:*** *Who are the sources of this information?*
    - ***Prompt:*** *Why do you use them?*
    - ***Prompt:*** *Are the sources of the information the same for human and veterinary drugs (if they sell both)?*
  - *Are you a member of a group or association related to your business?*
    - ***Prompt:*** *What do they do? WHAT ARE THE BENEFITS?*
    - ***Prompt:*** *Do they affect how you buy, sell or use antibiotics? How?*
  - *Who do you think are the people that you need to consult:*
    - *When you need to get a new supplier of antibiotics?*
    - *When you have doubts when selling/providing antibiotics to a client?*
    - *When understanding the regulations of antibiotics?*
  - *What are the major reasons for you to decide not to stock/prescribe/ advice any specific antibiotic? (probe for consequences- upstream, downstream and influencer level consequences)*

Local and financial governance

- - *What type of training do you get to work in this profession?*
    - ***Prompt:*** *Who give it to you? Why them?*
  - *Do you need any permission from civic body? If so, can you please describe the process of acquiring the permit*
    - ***Prompt: please describe how this work***
    - ***Prompt:*** *Was there any challenge?*
    - ***Prompt:*** *is there any support group or agent involved in procuring the permission,*
    - ***Prompt:*** *is there any costs involved and if so, are these acceptable/ reasonable?)*

**Key Topic 4: Validation of the map of the AB value chain** [ASK TO PARTICIPANTS TYPE I AND THOSE STAKEHOLDERS NOT ABLE TO PRODUCE A MAP OF THE VAUE CHAIN]

*INTERVIEWER TO SHOW AND EXPLAIN THE MAP OF THE CHAIN OBTAINED, AND THEN ASK:*

- *Do you agree with this representation of the chain?*
  - *Prompt: Why / Why not?*
  - *Prompt: Please explain what would you change this diagram? why?*
  - *Prompt: Where do you get antibiotics from, and who do you supply them to? Is it possible to quantify the proportion of flows?*
- *Are there any important types of people or node missing in this diagram?*
  - *If yes, which one? How would you incorporate it? What is their role in the system?*
  - *If yes, how would you differentiate these type of people?*
    - - *Probe: Differentiation based on:*
        - *method of operation,*
        - *size of operation*
        - *type of training or experience*
        - *type of clients served.*

**Key Topic 5: Quality of stakeholder’s antibiotic stock and advice to their client** [ASK TO ALL PARTICIPANTS]

- *What is the range of quality of antibiotics that you provide?*
- How do you differentiate the different quality of antibiotics in the system?
- ***Prompt:*** *What would make the antibiotic to be in the best quality category? Why?*
- ***Prompt:*** *What would make the antibiotic to be in the lower quality category? Why?*
- ***Prompt:*** *how do you categorize them? [Interviewer to draw in a blank sheet of paper the different type of qualities – as in diagram below]*
  - - ***Prompt:*** *What type of quality of antibiotic does this provider supply?[use the diagram to link providers to quality of antibiotics]*

QA

QB

QC

P1

P2

P3

- - - ***Prompt:*** *What quality of antibiotic do customers request or get? Why?[draw in the diagram the link of clients and quality]*

QA

QB

QC

P1

P2

P3

C1

C2

C3

- *How do you store the antibiotics at your enterprise? (Probe for tablets, capsules, syrups, and vial storage).*
- *What happens to the antibiotics after the end of their shelf life?*
  - ***Prompt:*** *is there any protocol in place? What is it? Or why not? If not, are they sold irrespective of sell by date?*
  - ***Prompt:*** *if disposed, how are these disposed? / If sold, to whom?*
- *What is your policy for restocking antibiotics? (probe: is it based on client demand? Cost vs profit? Shelf life? Perceived efficacy?)*

**Key Topic 6: Investigation of potential overlaps of human and animal antibiotic usage at household and farm level:**

- Usages of overlapping medical products- are *there any medical products that you use in both humans and animals?*
- ***Prompt:*** *if so which ones?*
- ***Prompt:*** *why do they use these for animals or humans?*
- ***Prompt:*** *in which situations would they use these products?*
- ***Prompt:*** *who provided these to you?*
- ***Prompt:*** *reasons why they used human antibiotics instead of animal antibiotics?*
- ***Prompt:*** *for what conditions/diseases /issues?*
- ***Prompt:*** *on whose advice? If this is from a provider, which provider?*

***[N.B. If possible can ask to show the drugs used in both humans and livestock again]***

***[If overlap in antibiotic use is identified]:***

- Usage of human antibiotics in livestock- *Can you describe the situation where this happened recently?*
- ***Prompt:*** *reasons why animal antibiotics were used instead of human antibiotics?*
- ***Prompt:*** *for what health problems/issues?*
- ***Prompt:*** *on whose advice? Which provider?*
- Usage of animal antibiotics in humans- *Can you describe the situation where this happened recently?*
- ***Prompt:*** *reasons why animal antibiotics were used instead of human antibiotics?*
- ***Prompt:*** *for what health problems/issues?*
- ***Prompt:*** *on whose advice? Which provider?*

*TO ALL*

- In your view, what are the main barriers for adequate use of antibiotic in the system?
  - Prompt: Why? (probe for patient expectations, cost, lack of enforcement, conflict of interests of drug suppliers/ prescribers, risk of food insecurity etc)
  - Prompt: What are the aspirations for better use of antibiotics?
  - Prompt: How can this be achieved? What are the main barriers to achieving this?
- In your view, what type of interventions do you think would be useful/effective to control or improve the use of antibiotics in the system?
  - Prompt: what would be a useful stewardship intervention? Why?
