# Supplementary material for: Pharma-cartography: Navigating the complexities of antibiotic supply to rural livestock in West Bengal, India, through value chain and power dynamic analysis
Source: PLoS One. 2023 Feb 2;18(2):e0281188. doi: 10.1371/journal.pone.0281188 (PMC9894437; doi:10.1371/journal.pone.0281188)
Supplement: S3 File — (DOCX) [file pone.0281188.s003.docx]

**A multi-stakeholder approach towards operationalising antibiotic stewardship in India’s pluralistic rural health system.**

**Mapping of supply and value chain**

**DRAFT Household Interview Guide - Antibiotic supply chain**

This guide will be used as a template for the semi-structured, qualitative interviews of livestock keeping households in/close to the site of investigation.

**Objective:** We aim to gain a better understanding of:

- The type of livestock systems present in the village with the objective of characterising these in this context
- How and why antibiotics are used in livestock in these systems and to ascertain their sources
- Potential overlap of usage and sources of human and animal antibiotics

Potential participants:

Households who keep livestock within the study site.

**Guidance for interviewers and note-takers**

**Introduction**

Before investigating the key topics of investigation, the researcher will provide an information sheet and discuss the project overview, the interviewer background, purpose and duration of the interview and provide the opportunity for questions. The researcher will then present the consent form for signing by the participant. The interview will proceed only once the consent form has been signed. Once the consent form is signed, the voice recorded should be activated.

**Before the interview, please ensure the following**

- You have gone through the interview guide and have familiarised yourself with the questions
- The recording device is working and is set up properly
- You have enough pages in your notebook to note the conversation, in case the provider does not consent to being recorded.

**At the beginning of the interview**

- Explain/read out all the required information about the research and the confidentiality issues before starting with the questions. Please use the information provided in the informed consent form.

**During the Interview**

- Try not to refer to the guide during the interview, but at the end make sure you have covered all the topics. Do not read out the questions.
- Ask the questions in a logical manner, and not necessarily in the same order as the topic guide. If the interviewee has already fully answered a particular question spontaneously do not ask the same question again just because it is the next question in the topic guide.
- Do not ask very sensitive questions in the beginning as this will make the interviewee suspicious. For example, do not ask questions about obtaining antibiotics without prescriptions at the beginning.
- Remain attentive and listen carefully to each answer. Do not interrupt when the interviewee is speaking unless the discussion is going into a totally irrelevant area.
- Before asking any question, think about how you are wording the question. Please do not ask leading questions. Keep them open ended and do not give the answer in your question. For example, instead of asking poultry farmers early on in the interview ‘Do you mix antibiotics in your chicken feed?’, ask ‘What are the ingredients that you mix in the feed for your chicken?’

**At the end of the interview**

- Make sure you obtained the interviewee’s signature on the consent form.
- Try to get some pictures, especially if the setting is interesting.
- If the pictures include any clearly visible and identifiable human subjects, it would be best to get their signed consent as this is required for any kind of publishing of photographs.

**Key Topic 1: Identification and Characterisation of the Livestock System:**

- Type, number and ownership of livestock- *what animals are reared? How many? Who owns the animals?*
- Purposes of rearing livestock- describe *the reasons why you keep livestock?* ***[Ensure reasons are captured for each species present]***
- ***Prompt:*** *for what products?*
- ***Prompt:*** *are any of your livestock kept for commercial/supplementary income/food security/cultural reasons (celebrations, festival or social status?)*
- ***Prompt:*** *how important is this activity for the household?*
- Feeding- *describe how your animals are fed?*
- ***Prompt****: what is fed (supplementary feed, scavenging, kitchen scraps) and why?*
- ***Prompt****: do you give them any growth promotors? If so what product did you give and to which animals? Why?*
- ***Prompt****: how do you get these products? Who do you get them from? Why these source?*
- Knowledge and understanding of livestock management- *explain to us how you learnt to look after your animals*
- ***Prompt:*** *how was knowledge about livestock management obtained?*
- ***Prompt:*** *What is their main source(s) of information for animal management?*

**Key Topic 2: Investigation of Antibiotic Usage, Drivers of Usage and their Sources in Livestock:**

- Management of livestock illness and livestock health seeking- *what do you do when one of your animals becomes sick? Can you describe a situation when this happened recently? What did you do? Why?* ***[Repeat question for each species of animal on the farm/household]***
- ***Prompt:*** *source of advice for care? Do you seek advice from a provider of health? For what reasons? Which ones? Why/why not?*
- ***Prompt:*** *who provides livestock treatment? Self-treatment? Why? When/at what point would they want to get treatment for their animals?*
- ***Prompt:*** *explore if approaches for the management of illness are different for different species of animals owned (if applicable) and why.*
- ***Prompt:*** *could you describe what was given to the animals as treatment?* ***[If possible identify antibiotic use or commercial name of the product]***
- Can you show us the medicines or product you have currently that are used in your livestock to improve health or performance?
  - Identify any antibiotic
  - Prompt: Is there other product you use that you do not have at the moment? If so, why one?
  - If antibiotic identified, prompt the questions below using these
- Understanding and awareness of antibiotics- *Can you explain to us what you know about antibiotics?*

***- Prompt:*** *do they understand what they are used for and how they work?*

***[N.B. may not understand what antibiotics are but may have been given/bought antibiotics- enquire if they understand how they work and probe for an alternative term for antibiotics (e.g. feed additive)]***

- Recent usage of antibiotics in livestock- [if product identified as antibiotic in previous question, ask about this product] *Describe the situation when antibiotics (or other term, or medication) were used last for one of your animals*
  - *If term antibiotic not know, then ask: describe medicine you use last time in your animals.*
    - *The following question can provide clues on how likely a product was an antibiotic. If no antibiotic, ask for another treatment or medicine used.*

***- Prompt:*** *What product was given? To which animals? Why?*

- ***Prompt*:** w*here and from whom were these obtained? Why?*

*-* ***Prompt****: on whose advice?*

***- Prompt:*** *is this a regular treatment option? How often do you give this to the animals? Why?*

***[N.B. can ask to show the drugs the household have in stock for the treatment of their livestock- ask how the drugs are used]***

- Reasons for antibiotic (or other term, or medication) usage- *for what reasons or problems would you use antibiotics for your animals?*
- ***Prompt:*** *Treatment/prophylaxis/growth promotion?*
- ***Prompt***: *failure of other methods such as ethno-veterinary/herbal/local treatments?*
- Accessibility to antibiotic (or other term or mediction) drugs- *how do you usually get antibiotics?*
- ***Prompt:*** *are there any barriers?*
- ***Prompt:*** *did anyone help you with advice for this? Who and why?*
- ***Prompt:*** *have you ever got antibiotics or drugs for your animals from your doctor? Why did you get them from them?*
- ***Prompt:*** *do you ever seek advice from your doctor regarding the health of your animals? Describe in which situations and why?*
- Administration of antibiotics- *Who administers the antibiotics to your animals?*
- ***Prompt:*** *by themselves or by the health provider?*

***Prompt:*** *are they aware of what has been given to them or administered by the health provider?*

- Understanding of antibiotic resistance - *have they observed treatment failure when using an antibiotic that usually was effective in curing their animals recently? Is this common? What do they do when this happens?*

**Key Topic 3: Investigation of Potential Overlaps of Human and Animal Antibiotic Usage at Household and Farm Level:**

- Usages of overlapping medical products- are *there any medical products that you use in both humans and animals?*
- ***Prompt:*** *if so which ones?*
- ***Prompt:*** *why do they use these for animals or humans?*
- ***Prompt:*** *when do they use these products?*
- ***Prompt:*** *who provided these to you?*
- ***Prompt****: reasons why they used human antibiotics instead of animal antibiotics?*
- ***Prompt:*** *for what conditions/diseases /issues?*
- ***Prompt:*** *on whose advice? If this from a provider, which provider?*

***[N.B. If possible can ask to show the drugs used in both humans and livestock again]***

***[If antibiotics are identified]***

- Perceptions of human and animal antibiotics- describe *what you understand to be the differences between human and animal antibiotics?*
- ***Prompt:*** *how they work?*
- ***Prompt:*** *in which situations would they be used for? Differences between people and animals?*
- Usage of human antibiotics in livestock- *Can you describe the situation where this happened recently?*
- ***Prompt:*** *reasons why animal antibiotics were used instead of human antibiotics?*
- ***Prompt:*** *for what health problems/issues?*
- ***Prompt:*** *on whose advice? Which provider?*
- Usage of animal antibiotics in humans- *Can you describe the situation where this happened recently?*
- ***Prompt:*** *reasons why animal antibiotics were used instead of human antibiotics?*
- ***Prompt:*** *for what health problems/issues?*
- ***Prompt:*** *on whose advice? Which provider?*
- Health seeking (livestock) - *can you explain which health providers you go to again for the treatment of your animals, if any? Why?*
- ***Prompt:*** *do these providers sometimes help you with some health issues of people in your household? When and how?*
- ***Prompt:*** *why do they go to the providers identified?*
- Health seeking (human) **–** *can you explain which health providers you go to for your own treatment or for the treatment of people in your household?*

*-* ***Prompt:*** *do any of these providers help you sometimes with some health problems of your animals? When and how?*

*-* ***Prompt:*** *Why do they go to the providers identified?*

*-*  ***Prompt:*** *is this for all their livestock or only for specific animals (e.g. cows versus chicken)? Why? If only treatment is only sought for specific species/systems, why is this?*

**Antibiotics that expire and interventions**

- *What happens to the antibiotics after the end of their shelf life?*
  - ***Prompt:*** *is there any protocol in place? What is it? Or why not? If not, are they sold irrespective of sell by date?*
  - ***Prompt:*** *if disposed, how are these disposed? / If sold, to whom?*
- In your view, what type of interventions do you think would be useful/effective to control or improve the use of antibiotics in the system?
  - Prompt: what would be a useful stewardship intervention? Why?
